# Supplementary material for: RNA m6a Methylation Regulator Expression in Castration-Resistant Prostate Cancer Progression and Its Genetic Associations
Source: Cancers (Basel). 2024 Mar 27;16(7):1303. doi: 10.3390/cancers16071303 (PMC11011207; doi:10.3390/cancers16071303)
Supplement: Supplementary file 1 [file cancers-16-01303-s001.zip › cancers-2853502-supplementary final.pdf]

# Supplementary Materials: RNA m6a Methylation Regulator Expression in Castration-Resistant Prostate Cancer Progression and its Genetic Associations

**Chamikara Liyanage, Achala Fernando, Audrey Chamberlain, Afshin Moradi and Jyotsna Batra**

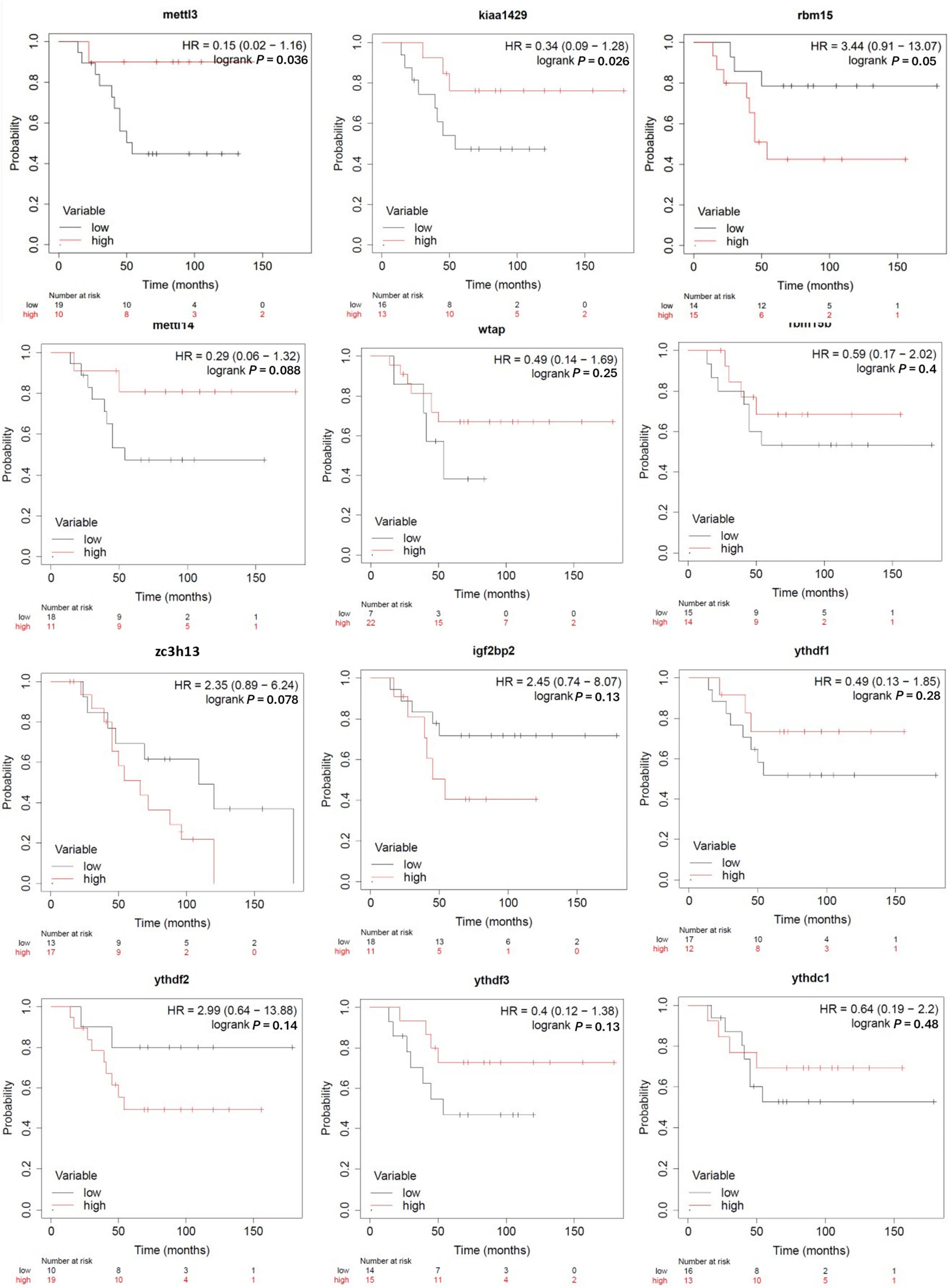
mettl3
HR = 0.15 (0.02 − 1.16)
logrank *P* = 0.036
kiaa1429
HR = 0.34 (0.09 − 1.28)
logrank *P* = 0.026
rbm15
HR = 3.44 (0.91 − 13.07)
logrank *P* = 0.05
HR = 0.29 (0.06 − 1.32)
logrank *P* = 0.088
wtap
HR = 0.49 (0.14 − 1.69)
logrank *P* = 0.25
HR = 0.59 (0.17 − 2.02)
logrank *P* = 0.4
zc3h13
HR = 2.35 (0.89 − 6.24)
logrank *P* = 0.078
igf2bp2
HR = 2.45 (0.74 − 8.07)
logrank *P* = 0.13
ythdf1
HR = 0.49 (0.13 − 1.85)
logrank *P* = 0.28
ythdf2
HR = 2.99 (0.64 − 13.88)
logrank *P* = 0.14
ythdf3
HR = 0.4 (0.12 − 1.38)
logrank *P* = 0.13
ythdc1
HR = 0.64 (0.19 − 2.2)
logrank *P* = 0.48
Probability
Time (months)
Variable
low
high
Number at risk

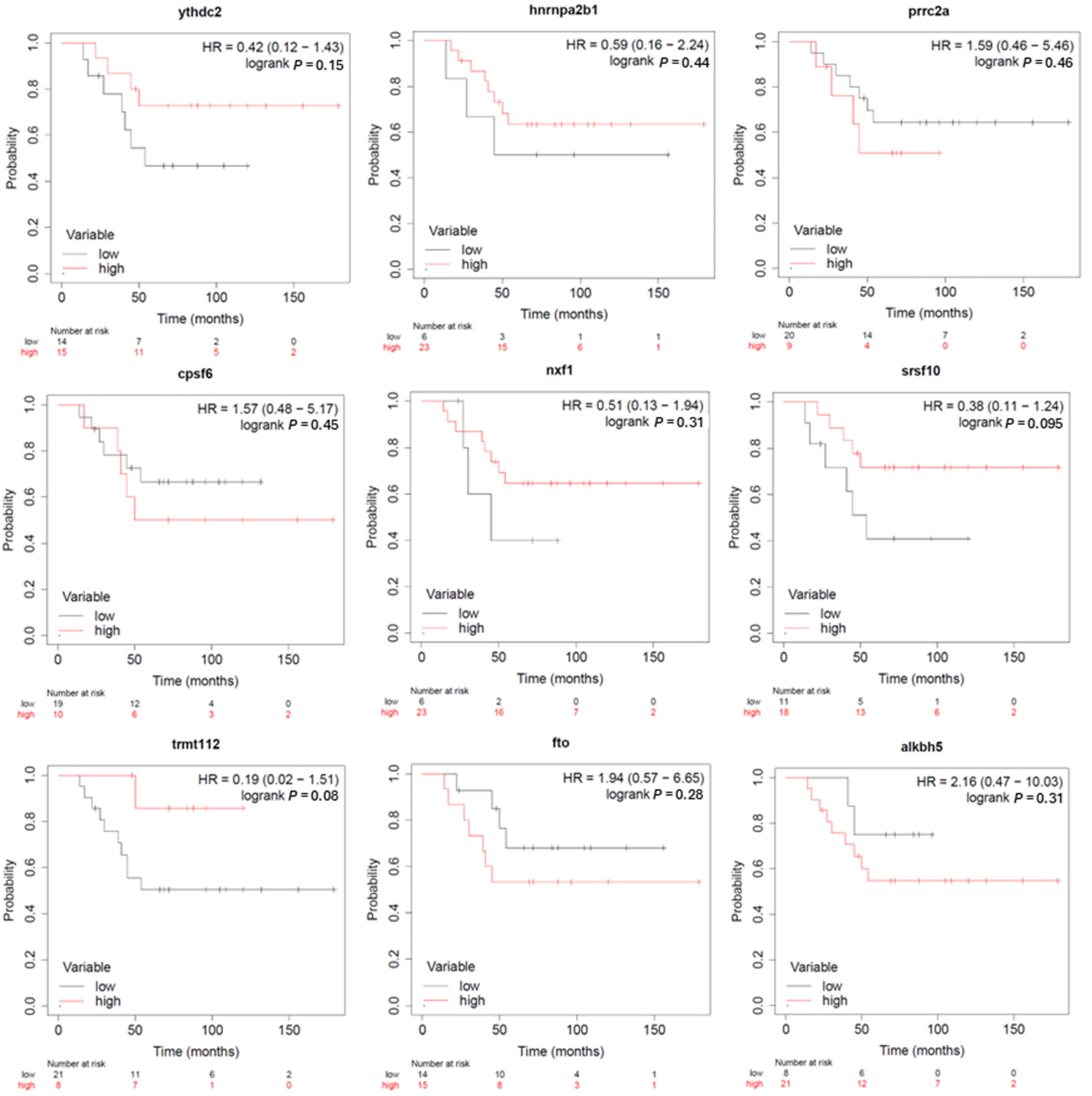


**Figure S1.** Univariate Kaplan-Meir Survival analysis of m6A methylation regulators for overall survival of CRPC patients after the first hormone refractory therapy. Based on the univariate cox regression analysis performed using the Kaplan-Meier Plotter, 18 m6A regulators showed no significant correlation between their expression and overall survival of CRPC patients. HR: hazard ratio; low: low expression of the m6A regulator; high: high expression of the m6A regulator.

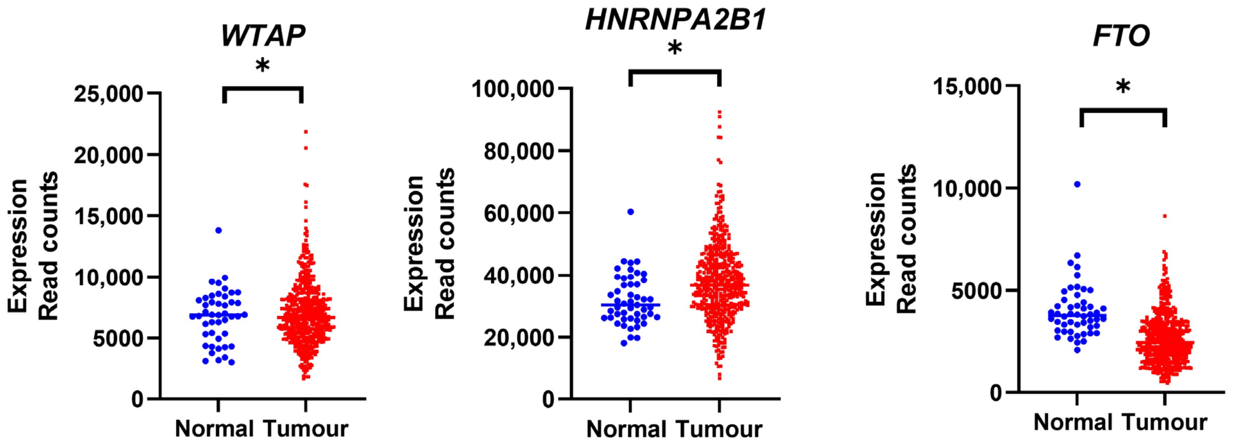


**Figure S2.** Three m6A regulator genes were found to be differentially expressed in primary PCa ($n$ = 450) tissue compared to healthy controls ($n$ = 49) using The Cancer Genome Atlas Program (TCGA) RNAseq data, * $p < 0.05$.

**Table S1.** SWATH-MS label-free quantification of m6A regulator expression in prostate cancer cell lines.

**Table S2.** SWATH-MS label-free quantification of m6A regulator expression in androgen- and anti-androgen-treated LNCaP cell line.

**Table S3.** RNA sequencing transcriptomic analysis of m6A regulator expression in androgen- and anti-androgen-treated LNCaP cell line.

**Table S4.** RNA sequencing analysis of m6A regulator expression in prostate cancer tumor and adjacent normal tissues.

**Table S5.** Log$_2$ median expression data for m6A regulatory genes recruited from the Grasso dataset (N = Normal Tissue, T = Localized Prostate Cancer, WA = CRPC).

**Table S6.** Survival data of CRPC patients after first hormone therapy from the Grasso dataset (survival event at 5 years 1 = Dead, 0 = Alive).

**Table S7.** PCa risk loci association with m6A regulator genes.

**Table S1–S7.** are attached separately as an Excel file.
